# Censorship of the Medical Press

**Published:** 1907-05

**Authors:** 


### EDITORIAL DEPARTMENT.

### CENSORSHIP OF THE MEDICAL PRESS.

CZAR METHODS OF THE OCTOPUS.

Amongst the "Regulations Regarding Exhibits" at this year's meeting of the A. M. A. is the following:

"No medical journal or publication can be exhibited that contains advertisements of drug, chemical or similar preparation used in the treatment of disease, which does not conform to the rules of the Council on Pharmacy and Chemistry of the American Medical Association."

The enforcement of this ukase will exclude practically every medical journal published in America and Canada, excepting the "State" journals,—those wearing the collar of the octopus and bearing the union stamp,—and it will, or should, include the California tentacle also.

Are we living in America, or in darkest Russia? Is this the fourteenth or the twentieth century? Are we freemen, or serfs? Have we—as owners and publishers of legitimate medical journals—any rights whatever, that the machine is bound to respect? Have we the right to think and speak our sentiments—or are we to be controlled, in both, by the monstrous machine which would dictate to us, and "muzzle the press," under the denunciation of being "unclean," "infectious" and unworthy of support by the profession? Shall we not be permitted "to differ with Brother Paul"—as to the respectability of a pharmacal preparation? Who gave this council the right to dictate what the press shall and shall not advertise? This is the most insolent, high-handed and outrageous act, of all the brazen acts, of the political machine that controls the A. M. A. and its "organ." Will the effrontery and arbitrary exercise of a self-assumed authority, on the part of the Octopus, stop here? Hardly. If this is submitted to by the American medical editors—those who do not wear the collar and the union stamp—we may look, next, to see an inspector with the A. M. A. union badge, stationed at each door at future meetings, to search our persons to see if any one has concealed about him a copy of any of the "infectious" and "unclean" publications (as Chase and Jonesey call the independent journals). We may look to see such person arrested, caught in the act of smuggling contraband publications, and arraigned on a charge of treason to the autocrat,—

the czar of all the "schools" of medicine. Shall we, like Socrates, for "corrupting the youth of Athens," be made to quaff the hemlock for corrupting the morals of the younger men of medicine by insisting that there is virtue in pharmacy by the large as well as per the obsolete prescription compounding, and that thousands of the most experienced use,—and find useful,—hosts of the preparations advertised in the medical press? Will our editorial utterances be censored next?

The Association of American Medical Editors will meet at Atlantic City, June 4th, simultaneously, with the A. M. A. We will see what they will do about this monstrous assumption of authority. If they tamely submit to the thraldom—submit to being put in irons, as it were, and exhibited in the pillory as "unclean and infectious" and unfit to be represented in the A. M. A., they may next expect to be bodily kicked out, and they would deserve to be. These editors are all members of the A. M. A., and pay tribute to Cæsar.

I reproduce the following from the Texas tentacle of the Octopus:

" * * * A careful perusal of the reading pages of most of these journals will demonstrate a bitter, prejudicial, carping, vindictive and antagonistic spirit on many vital subjects affecting modern progress. These journals might do a useful work; we wish them well, *but they must clean house or be labeled 'infectious,' and be shunned by the profession."—Abstract from an editorial in the Texas State Journal of Medicine,* March, 1907.

["Modern progress" is good. Progress, *backward,* like the crab. It is a new name for degeneracy.—D.]

I will say for myself, I am not a member of the A. M. A., having drawn out fifteen years ago,—although I have been solicited—by Simmons—many times, to become a member. I am not in accord with the policy of the clique who control the Texas State Medical Associatión; who have played that splendid body into the hands of the Octopus, and who would now out-Herod Herod! I am "bitter and antagonistic" only to the trickery which has enabled certain pliant and complaisant members to get control of the Association, and to gag and tie the majority. I am fighting for the Association, to free it from this thraldom; and because I love the Association and grieve to see it so debased. I have, always, for twenty odd years, stood for, advocated, championed, and defended the best interests of the body of the Association, as against those who would degrade it, and who, by the forcing down our throats of that absurd Joseph's coat of many colors—the absurd mixed board—have degraded it. Two of our most distinguished leaders, and ex-Presidents, denounced it, to me, as a disgrace to the profession, and a stultification. Both these gentlemen—who, for twenty-five years, have not missed a meeting—absented themselves, as did yours truly, from the recent meeting at Mineral Wells. The political methods of the minority will drive off many other self-respecting members. They will surrender with a silent protest, and will stay away. I go on record, for the hundredth time, perhaps, as protesting with all my soul against the recognition of the pathies as "brethren,"—the recognition of and toadying to these people whom the "regulars" have, for years, snubbed and ridiculed. I'll die with my boots on and my spurs, if die I must, fighting for the rights and the honor of legitimate medicine.

I know that in doing so I antagonize some warm personal friends; but it is not a matter of friendship,—it is a matter of principle;—and I have the consolation of knowing, feeling and believing that I am *right;* though my "policy" may not be "expedient," nor fit in with what the fanatics in control call the "reform" movement; "reform" inaugurated by a reformed homeopath, who, by the irony of Fate, becomes the leader and dictator of regular medicine!

Will ye editors wear his collar?

